# ﻿Mitochondrial genome data provide insights into the phylogenetic relationships within *Triplophysadalaica* (Kessler, 1876) (Cypriniformes, Nemacheilidae)

**DOI:** 10.3897/zookeys.1197.116342

**Published:** 2024-04-03

**Authors:** Hao Meng, Yingnan Wang, Ge-Xia Qiao, Jun Chen

**Affiliations:** 1 Key Laboratory of Zoological Systematics and Evolution, Institute of Zoology, Chinese Academy of Sciences, Beijing 100101, China Institute of Zoology, Chinese Academy of Sciences Beijing China; 2 National Animal Collection Resource Center, Institute of Zoology, Chinese Academy of Sciences, Beijing 100101, China Institute of Zoology, Chinese Academy of Sciences Beijing China

**Keywords:** High-throughput sequencing, mitogenome assembly, phylogeny, stone loach

## Abstract

Due to the detrimental effect of formaldehyde on DNA, ethanol has replaced formalin as the primary preservative for animal specimens. However, short-term formalin fixation of specimens might be applied during field collection. In an increasing number of studies, DNA extraction and sequencing have been successfully conducted from formalin-fixed specimens. Here the DNA from five specimens of *Triplophysadalaica* (Kessler, 1876) were extracted and performed high-throughput sequencing. Four of the specimens underwent short-term fixation with formalin and were subsequently transferred to ethanol. One was continuously stored in ethanol. No significant difference of DNA quality and amount were observed among these samples. Followed by assembly and annotation, five mitochondrial genomes ranging in length from 16,569 to 16,572 bp were obtained. Additionally, previously published data of other individuals or species were included to perform phylogenetic analyses. In the reconstructed trees, all eight individuals of *T.dalaica* form a monophyletic group within the *Triplophysa* branch. The group is divided into three clades: (1) samples from the Yellow River, (2) those from the Yangtze River, and (3) those from the Haihe River, and the Lake Dali Nur. This study sheds initial light on the phylogeographic relationships among different populations of *T.dalaica*, and will support the research about its evolutionary history in the future.

## ﻿Introduction

Biological specimens preserved in museums represent a reservoir of valuable data. They provide information required in various fields such as taxonomy, geographic distribution, population dynamics, and climate change (Suarez and Tsutsui 2004; Wen et al. 2015). With the progression of historic DNA (hDNA) extraction and sequencing, genetic material contained in specimens of varying ages, taxa, and preservation methods have been more adequately explored (Hartley et al. 2006; Hartnup et al. 2011; Tin et al. 2014; Blaimer et al. 2016; Hawkins et al. 2016; Kehlmaier et al. 2019; Lalonde and Marcus 2020; Cong et al. 2021).

Formalin, an aqueous solution of formaldehyde, has been widely used as a preservative for specimens of invertebrates, fish, amphibians, and reptiles. However, it is a formidable challenge to extract hDNA from formalin-fixed specimens due to formalin’s propensity to induce three forms of DNA damage in specimens: (1) fragmentation, (2) cross-linking between DNA and protein molecules, and (3) modification of DNA bases (Quach et al. 2004; Campos and Gilbert 2012; Do and Dobrovic 2012; Wong et al. 2014). In recent studies, hDNA in formalin-fixed specimens have been successfully sequenced (e.g. Hykin et al. 2015; Ruane and Austin 2017; Silva et al. 2017; Kehlmaier et al. 2020; Appleyard et al. 2021; Straube et al. 2021; Agne et al. 2022; Bernstein and Ruane 2022; O’Connell et al. 2022). Most of the modified extraction protocols focus on removal of residual formaldehyde and digestion. The approaches include replacing formaldehyde with ethanol at varying concentrations, pre-treating with high temperature, adding extra proteinase K, and extending digestion time.

Owing to the deleterious impact on genetic material and the inherent toxicity of formaldehyde, ethanol has been used as a preferred preservative instead of formalin. An increasing number of museums have transferred historical specimens preserved in formalin to ethanol. Moreover, due to limitations during fieldwork, specimens may undergo a temporary fixation in formalin and later transferred to ethanol for preservation. This study attempts to process these formalin-to-ethanol samples and conduct high-throughput sequencing (HTS).

*Triplophysa* is one of the most diverse genera within the family Nemacheilidae, with over 140 documented species in FishBase (Froese and Pauly 2023). In China, species of *Triplophysa* are predominantly distributed across the Qinghai-Tibet Plateau and adjacent regions, with a few occurrences in North China (Zhu 1989). Due to their widespread distribution, species of *Triplophysa* exhibit adaptability to diverse habitats, including cold environments, hypoxia, and saline waters. In recent years, multiple aspects including the phylogenetic relationships among *Triplophysa* species (Wang et al. 2016, 2023; Chen et al. 2019; Wu et al. 2020), phylogeography (Hu et al. 2020, 2022; Zhong et al. 2022; Du et al. 2023), patterns of population differentiation (Zhou et al. 2021b; Jin et al. 2022), and the adaptations to various habitats (Chen et al. 2020b, 2020a; Zhang et al. 2023) have been studied.

Within the genus, *Triplophysadalaica* (Kessler, 1876) is an endemic species in China. It was initially described and collected from the Lake Dali Nur, which is an alkaline lake located in Inner Mongolia, China (43.38°N, 116.72°43′E). In addition to the Lake Dali Nur and its surrounding lakes and rivers, *T.dalaica* is also distributed in fresh water such as the Yellow River (Zhu 1989; Zhou et al. 2021a), the Haihe River (Zhu 1989; Wang et al. 2001; Zhou et al. 2020), and the Yangtze River (Chen et al. 1987). Recently, a chromosome-level reference genome of the *T.dalaica* was assembled using a sample from the Lake Dali Nur (Zhou et al. 2021a). Other research has primarily focused on its adaptation in response to factors such as hypoxia and salinity fluctuations (Wang et al. 2015; Zhou et al. 2022). Moreover, several analyses related to the phylogeny of loaches within the family Nemacheilidae or the genus *Trioplophysa* have included this species (Wang et al. 2016, 2023; Chen et al. 2019; Wu et al. 2020). Nevertheless, the genetic relationships among populations from different water systems remain unknown.

Current study involves five mitogenomes obtained through HTS from *T.dalaica* specimens, and three *T.dalaica* mitogenomes published or assembled from released HTS data, along with 16 published mitogenomes of other species as outgroups. The analyses aim to confirm the taxonomic status of *T.dalaica*, and reconstruct the phylogenetic relationship of populations residing different geographic origins.

## ﻿Materials and methods

### ﻿Sample selection and DNA extraction

**Figure 1.**
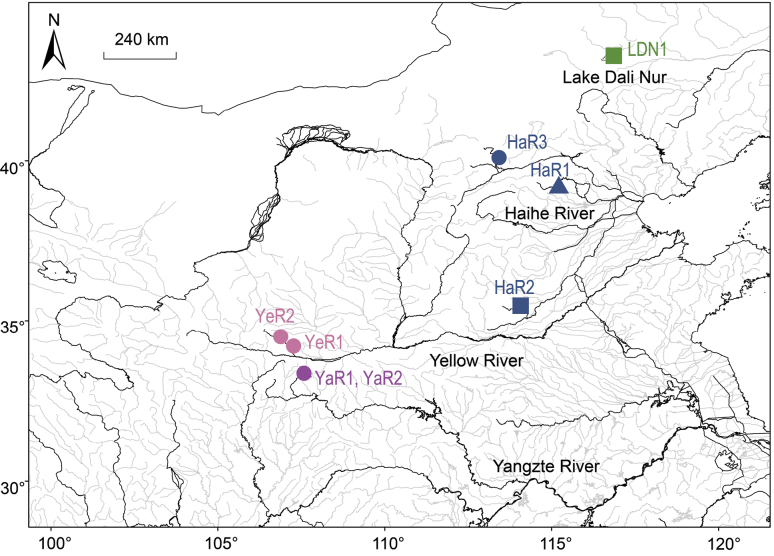
Geographic distribution of the eight *Triplophysadalaica* samples. Circles: samples sequenced in this research. Triangle: sample with mitochondrial genome available in NCBI. Squares: samples for which mitochondrial genome assembled using released HTS data from NCBI.

In this study, five specimens of *Triplophysadalaica* were selected from the National Animal Collection Resource Center, representing individuals originating from three distinct rivers (Fig. 1; Table 1). Among these, four individuals collected in 2013 underwent formalin fixation for approximately 30 days, while one individual collected in 2019 has been continuously preserved in ethanol with a concentration exceeding 90%. These specimens were chosen to investigate the effects of short-term formalin fixation on the quality and suitability of the preserved material for molecular experiments.

**Table 1.** Information of the eight *Triplophysadalaica* samples.

ID	Location	Water System	Data Acc. No.	Source
YeR1	34.66°N, 107.04°E	Yellow River	OR857523	This study
YeR2	34.94°N, 106.72°E	Yellow River	OR857524	This study
YaR1	33.85°N, 107.46°E	Yangtze River	OR857525	This study
YaR2	33.85°N, 107.46°E	Yangtze River	OR857526	This study
HaR3	40.32°N, 113.30°E	Haihe River	OR857527	This study
HaR1	Hebei Province	Haihe River	KY945353	Submitted by Feng et al.
HaR2	35.91°N, 113.86°E	Haihe River	SRX8097844	Zhou et al. 2021a
LDN1	43.38°N, 116.66°E	Lake Dali Nur	SRX8097848	Zhou et al. 2021a

To minimize the potential contamination, the entire DNA extraction process was conducted in a laboratory that had not previously been exposed to fish samples. Fin clips, approximately 5 mm in length from the tip of the right pectoral fin, were utilized. For formalin-fixed samples, a modified version of the protocol outlined by a previous study (Xia et al. 2007) was employed to pre-treat the samples for the removal of any residual formalin. Following the pre-treatment steps, the DNA was extracted using the DNeasy Blood & Tissue Kit (Qiagen, Shanghai, China) and the MinElute PCR Purification Kit (Qiagen, Shanghai, China) to maximize the recovery of fragmented DNA fragments. The protocols for DNA extraction have been uploaded to protocols.io (https://www.protocols.io/private/FE16637289CE11EE83B20A58A9FEAC02). The concentration and fragmentation of DNA were assessed by agarose gel electrophoresis or the Agilent 5400 system (Agilent, Palo Alto, USA).

### ﻿High-throughput sequencing data acquisition

High-throughput sequencing (HTS) was conducted on a Illumina platform with PE150 strategy at Berry Genomics (Beijing, China) and Novogene Bioinformatics Technology Co., Ltd (Beijing, China). In addition to the five individuals sequenced for this study, HTS data for two *T.dalaica* individuals from the Lake Dali Nur and the Haihe River (Fig. 1; Table 1) were obtained from Sequence Read Archive (SRA) on the National Center for Biotechnology Information (NCBI) database using the SRA toolkit v 3.0.5 (https://github.com/ncbi/sra-tools/). Approximately 40 million reads were extracted from each individual for subsequent assembly of mitochondrial genomes.

The reads were subjected to quality control using fastp v. 0.23.4 (Chen et al. 2018; Chen 2023). This process involved the removal of reads with an overall quality score lower than 20, elimination of redundant duplicate reads, and trimming of adapter fragments from both ends.

### ﻿Mitogenome assembly and annotation

The reads were mapped to the reference mitogenome of *T.dalaica* in NCBI (accession number KY945353) using Geneious v. 9.1.8 (Biomatters Ltd, Auckland, New Zealand) with 10 iterations in medium-low sensitivity. To eliminate nuclear mitochondrial DNA segments (NUMTs), the mapped reads were subjected to *de novo* assembly using Geneious assembler. This approach yielded a contig approximately 16 kb in length. Subsequently, a manual inspection and sequence concatenation process was performed at both ends of the contig, resulting in the circular mitochondrial genome.

Following the assembly, the mitogenome was annotated on GeSeq (Tillich et al. 2017) using mitochondrial genomes of *Triplophysa* species available on NCBI as reference sequences. The annotation results also underwent rigorous manual verification.

### ﻿Phylogenetic reconstruction

In addition to the five mitogenomes obtained in our study and the two assembled from published HTS data, an additional mitogenome of *T.dalaica* from the Haihe River (Fig. 1; Table 1, Suppl. material 1: tables S2, S3) was downloaded from NCBI. Published mitogenomes from another 13 species of *Triplophysa*, two species of Cobitidae, and one species of Gastromyzontidae were also included as outgroups in the phylogenetic analysis (Suppl. material 1: table S3).

The sequences for 13 protein-coding genes (PCGs) without stop codon from the mitogenomes were extracted and aligned based on their translated amino acid sequences with Geneious v. 9.1.8. These alignments were concatenated and indels were preserved, resulting in a total alignment length of 11,427 bp. Subsequently, the optimal partitioning scheme and substitution model for different genes and codon positions were determined by PartitionFinder 2 (Lanfear et al. 2012). Based on 13 PCGs and the positions of three codons, the alignment was partitioned into 39 segments. A “greedy” algorithm was employed to determine the best-fitting partition scheme, estimated by the Bayesian information criterion (BIC). At the same time, the optimal nucleotide substitution model for each partition was determined.

The phylogenetic analysis was performed using both maximum-likelihood (ML) and Bayesian-inference (BI) methods based on the best-fitting partition strategy. For the ML analysis, 1,000 fast bootstrap replicates were conducted with the GTR+I+G substitution model to assess the support values using RAxML v. 8.2.12 (Stamatakis 2014). The BI analysis was conducted using MrBayes v. 3.2.6 (Ronquist et al. 2012). For each partition, the best-fitting model estimated by PartitionFinder 2 was applied. The analysis was performed with four independent runs, each comprising 50 million generations, and the tree was sampled every 1,000 generations. Then the average standard deviation of split frequency and Tracer v. 1.7.2 software was used to assess the convergence of the BI analysis. After discarding the first 12,500 trees as conservative burn-in, the final consensus tree was obtained, and branch-support values were evaluated using Bayesian posterior probabilities (BPP) under a majority-rule criterion.

### ﻿Divergence time estimation for the three *T.dalaica* clades

With the alignments of the 13 PCGs, the divergence time for three main clades of *T.dalaica* was estimated by a Markov chain Monte Carlo (MCMC) approach using BEAST v. 2.7.6 (Bouckaert et al. 2019). The Yule model was set as tree prior and the random local clock model was used. The nucleotide substitution model was set as suggested by PartitionFinder 2.

Because there is no solid fossil record of *Triplophysa*, a fossil of the genus *Cobitis* was used as time calibration for the tree (Suppl. material 1: fig. S1). The fossil was estimated to be 13.8–15.9 Ma (Zhou 1992). Three parallel runs were conducted with 100 million generations and the trees were logged every 1,000 generations. The convergence of the parameters was assessed by Tracer v. 1.7.2 (Rambaut et al. 2018) with ESS value. The maximum clade credibility tree was obtained by TreeAnnotator v. 2.7.6 (Bouckaert et al. 2019).

## ﻿Results

DNA was successfully extracted from the specimens of five *Triplophysadalaica*. Agarose gel electrophoresis and fragment analysis by Agilent 5400 revealed varying degrees of degradation in the DNA from all these samples, with most fragments shorter than 4 kb. When utilizing nearly equal amounts of fin tissue samples, the DNA concentrations obtained from all five samples fell within the range of 10–30 ng/μL (Suppl. material 1: table S1).

High-throughput sequencing of the five samples yielded an average of approximately 5 Gb of data per sample (Suppl. material 1: table S2). Following quality control and *de novo* assembly, the lengths of mitochondrial genomes ranged from 16,569 to 16,572 bp, and the depth coverage spanning from 16.8-fold to 275.9-fold. Additionally, from published SRA files from two *T.dalaica* individuals collected in the Lake Dali Nur and Haihe River, 36–40 million reads were selected for each individual. After quality control and *de novo* assembly, these reads resulted in two mitochondrial genomes of 16,569 bp in length, with depths of 821.9-fold and 347.8-fold, respectively. Furthermore, a mitogenome for a *T.dalaica* sample from the Haihe River was downloaded from NCBI, which length is 16,569 bp.

The above eight mitochondrial genomes displayed an average base composition of A: 28.13%, T: 28.14%, G: 18.04%, C: 25.63%, and the GC content was 43.67%. These compositions exhibited no significant differences among each other (Chi-squared test, df = 6, *p* = 0.634). Following *de novo* assembly, seven mitochondrial genomes were annotated with GeSeq. These mitochondrial genomes included 22 transfer RNA (tRNA) genes, two ribosomal RNA (rRNA) genes, 13 protein-coding genes (PCGs), and one non-coding control region. The 13 PCGs in these eight *T.dalaica* mitochondrial genomes spanned 11,421 bp (including stop codons), encoding a total of 3,800 amino acids. Among these, 1,754 nucleotide and 149 amino acid variable sites were observed. All nucleotide mutations were found to be substitutions, and no insertions or deletions were detected (Suppl. material 1: table S4).

The coding sequences (CDS) without stop codon of the 13 mitochondrial PCGs from these eight *T.dalaica* individuals, along with 13 other *Triplophysa* species, two Cobitidae species, and one Gastromyzontidae species, yielded a total alignment with length of 11,427 bp. This alignment was divided into 39 user-defined partitions based on the three different codon positions of the 13 PCGs. The best scheme consisted of four partitions with its own best-fitting substitution model according to PartitionFinder 2 (Suppl. material 1: table S5).

**Figure 2.**
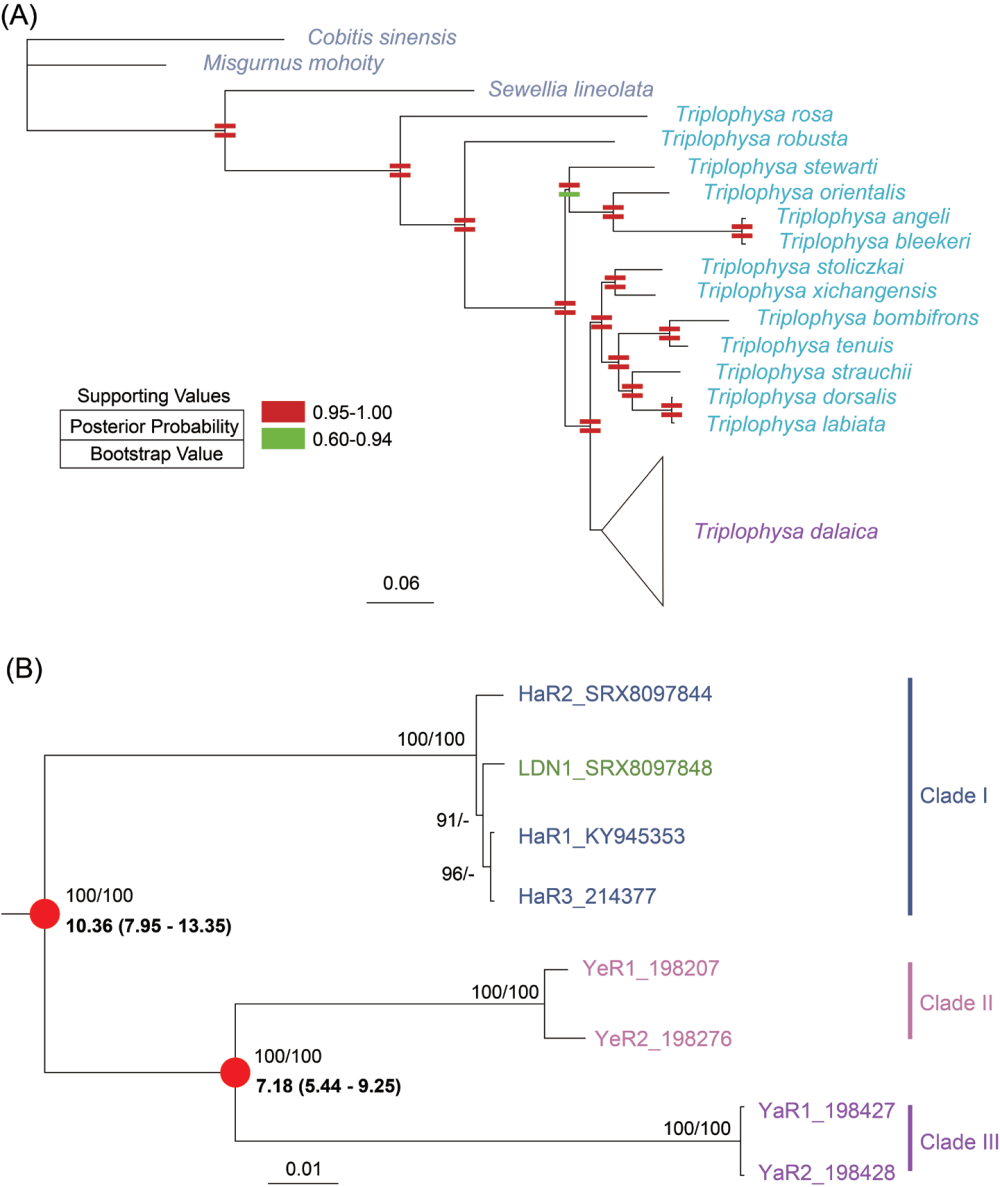
**A** The majority rule consensus tree constructed using MrBayes based on the CDS of 13 mitochondrial PCGs (excluding stop codons) of eight *Triplophysadalaica* individuals and outgroup species, totaling 11,427 bp. The topology of the tree closely resembles that constructed by RAxML. Posterior probabilities (from MrBayes) and bootstrap values (from RAxML) for branches are depicted as two different colored rectangles, one above the other **B** details of the clade containing the eight *T.dalaica* individuals in the phylogenetic tree. Numerical values on branches represent posterior probabilities and bootstrap values, respectively. The dashes represent values less than 50. The red dots indicate divergence time estimated by MCMC approach with 95% HPD.

Subsequently, employing the best-fitting partitioning scheme, phylogenetic trees for all 24 taxa were reconstructed using both RaXML (with a unified GTR + I + G model) and MrBayes (with individual partition-specific best-fitting substitution models). The resulting majority rule consensus trees (Fig. 2) exhibited nearly identical topology. In the trees, all *T.dalaica* individuals clustered together in a well-supported clade, sharing the same branch with other *Triplophysa* species (Fig. 2A).

Within the branch of *T.dalaica* (Fig. 2B), three distinct lineages were observed: (I) individuals from the Yangtze River (YaR1 and YaR2); (II) samples from the Yellow River (YeR1 and YeR2); and (III) individuals from the Lake Dali Nur (LDN1) and the Haihe River (HaR1, HaR2, and HaR3). Furthermore, individuals from the Yangtze River and the Yellow River are reciprocally sister groups. Within clade III, the individuals from disconnected water systems did not exhibit significant genetic differentiation.

The divergence time between the *T.dalaica* and its sister clade containing *T.dorsalis* and *T.stoliczkai* was estimated at 11.35 Ma (95% HPD: 8.75–14.6 Ma; Suppl. material 1: fig. S1), which is close to the time estimated by previous studies (Wang et al. 2016; Wu et al. 2020). Within *T.dalaica*, the divergence time between clade I and MRCA of clades II and III was estimated at approximately 10.36 Ma (95% HPD: 7.95–13.35 Ma), and the clades II and III diverged at about 7.18 Ma (95% HPD: 5.44–9.25 Ma).

## ﻿Discussion

In this study, DNA was extracted from four samples that underwent short-term formalin fixation and one sample continuously preserved in ethanol. Notably, there were no significant differences in DNA concentration or fragmentation among these samples, and mitogenomic sequences were successfully assembled. It suggests that short-term formalin fixation (for around 30 days) may not significantly contribute to DNA degradation. Therefore, when ethanol is unavailable due to acquisition or transportation in the field, short-term formalin fixation may be considered an acceptable approach. However, prolonged immersion in formalin would lead to irreversible DNA damage. (Quach et al. 2004; Campos and Gilbert 2012; Do and Dobrovic 2012; Wong et al. 2014). It is advisable to promptly transfer samples into preservation solutions like ethanol following the completion of sampling, to mitigate any potential long-term DNA degradation.

The *Triplophysadalaica* has been reported to inhabit the Lake Dali Nur, surrounding rivers and lakes of Inner Mongolia, as well as the Yellow River, the Haihe River, and the Yangtze River. The geographic sources of the samples analyzed in this study encompass all four of mentioned regions. All *T.dalaica* individuals form a monophyletic branch. This suggests that these eight individuals are the same taxon which belongs to the genus *Triplophysa*. Within the clade, the individuals from the Yangtze River (YaR1 and YaR2) and the Yellow River (YeR1 and YeR2) form two well-supported clusters, signifying the genetic distinctiveness of these two populations. They exhibit a sister relationship, suggesting their most common ancestor diverged from the clade III. Additionally, the sampling sites of the Yangtze and the Yellow Rivers are geographically adjacent, with a straight-line distance of approximately 100 km on the map. Their closer spatial distance correlates their genetic distance.

Clade III includes individuals from the Haihe River (HaR1, HaR2, and HaR3), and the Lake Dali Nur (LDN1), indicating close relationships among these individuals. The divergence-time estimation with mitochondrial PCGs also suggested they differentiated recently. However, a prior study conducted demographic history analysis using whole-genome resequencing data with the software PSMC for these two individuals, and the change of effective population size implied that populations from the Lake Dali Nur and the Haihe River might diverge approximately 1 million years ago (Zhou et al. 2021a). The inconsistency might be caused by mitochondrial introgression or another process. To address the evolutionary history of these two populations, more individuals from Lake Dali Nur and the Haihe River should be collected, and a broader set of nuclear genomic loci should be employed.

In the divergence-time analysis, these three clades were estimated to diverged 7–10 Ma. The divergence is relatively deep compared to the intraspecies differentiation in other *Triplophysa* species (Hu et al. 2020, 2022; Wu et al. 2020; Du et al. 2023), suggesting they might be divided into several potential subspecies, or even species, which need extra analyses including morphology and phylogeny with more samples. These results offer insights into the evolutionary history of *T.dalaica*, as well as the correlation between the change of population and historical climate and geological events.

## ﻿Additional information

### Author ORCIDs

Hao Meng https://orcid.org/0000-0002-9672-9706

Jun Chen https://orcid.org/0000-0002-0325-2532

### Data availability

All of the data that support the findings of this study are available in the main text or Supplementary Information.

## Supplementary materials

Supplementary material 1 Supplementary data Data type pdf Explanation note **table S1.** Collecting and extraction information of the five *Triplophysadalaica* samples; **table S2.** Summary of reads used for the mitochondrial genome assembly; **table S3.** Summary of the published mitogenomes used in phylogenetic analyses; **table S4.** Variable sites of the 13 PCGs in eight *T.dalaica* mitochondrial genomes; **table S5.** The best partition scheme and substitution model by PartitionFinder 2; **figure S1.** Divergence time among mitogenomes in this study with MCMC approach. https://binary.pensoft.net/file/1014336This dataset is made available under the Open Database License (http://opendatacommons.org/licenses/odbl/1.0/). The Open Database License (ODbL) is a license agreement intended to allow users to freely share, modify, and use this Dataset while maintaining this same freedom for others, provided that the original source and author(s) are credited.Hao Meng, Yingnan Wang, Ge-Xia Qiao, Jun Chen
