# Supplementary material for: ﻿Mitochondrial genome data provide insights into the phylogenetic relationships within Triplophysadalaica (Kessler, 1876) (Cypriniformes, Nemacheilidae)
Source: Zookeys. 2024 Apr 3;1197:43–55. doi: 10.3897/zookeys.1197.116342 (PMC11015090; doi:10.3897/zookeys.1197.116342)

Supplementary data:

Mitochondrial genome data provide insights into the phylogenetic relationships within *Triplophysa dalaica* (Kessler, 1876) (Cypriniformes, Nemacheilidae)

Hao Meng[1], Yingnan Wang[2], Ge-Xia Qiao[1,2], Jun Chen[1,2]

1 *Key Laboratory of Zoological Systematics and Evolution, Institute of Zoology, Chinese Academy of Sciences, Beijing 100101, China*

2 *National Animal Collection Resource Center, Institute of Zoology, Chinese Academy of Sciences, Beijing 100101, China*

Corresponding author: Ge-Xia Qiao (*qiaogx@ioz.ac.cn*), Jun Chen (*chenj@ioz.ac.cn*)

Table S1. Collecting and extraction information of the five *T. dalaica* samples.

Table S2. Summary of reads used for the mitochondrial genome assembly.

Table S3. Summary of the published mitogenomes used in phylogenetic analyses.

Table S4. Variable sites of the 13 PCGs in eight *T. dalaica* mitochondrial genomes

Table S5. The best partition scheme and substitution model by PartitionFinder 2.

Fig S1. Divergence time among mitogenomes in this study with MCMC approach.

Table S1. Collecting and extraction information of the five *T. dalaica* samples.

**ID**	**Voucher ID**	**Location**	**Longitude**	**Latitude**	**Sample Date**	**Collector**	**Water System**	**Formalin-fixed Duration**	**DNA Concentration (ng/μL)**
YeR1	198207	Baoji, Shaanxi	107.04°E	34.66°N	2013/8/1	Chunguang Zhang and Yingchun Xing	Yellow River	About 30 days	25.775
YeR2	198276	Baoji, Shaanxi	106.72°E	34.94°N	2013/8/1	Chunguang Zhang and Yingchun Xing	Yellow River	About 30 days	26.222
YaR1	198427	Baoji, Shaanxi	107.46°E	33.86°N	2013/3/26	Chunguang Zhang and Yingchun Xing	Yangtze River	About 30 days	16.334
YaR2	198428	Baoji, Shaanxi	107.46°E	33.86°N	2013/3/26	Chunguang Zhang and Yingchun Xing	Yangtze River	About 30 days	20.498
HaR3	214377	Datong, Shanxi	113.30°E	40.32°N	2019/10/15	Haipeng Liu, Xuejian Li, Chengyi Niu and Jie Bai	Haihe River	Untreated with formalin	25.957

Table S2. Summary of reads used for the mitochondrial genome assembly.

ID	Clean reads	Clean bases (bp)	Mapped reads	Mapped bases (bp)	Reads for assembling	Bases for assembling (bp)	Mitogenome size (bp)	Mean coverage	Source
YeR1	33,509,836	4,972,189,717	16,593	2,475,855	16,570	2,455,928	16,572	148.2	This study
YeR2	33,297,566	4,961,015,853	30,650	4,614,069	30,474	4,571,998	16,572	275.9	This study
YaR1	12,962,500	1,902,712,394	1,971	296,321	1,955	277,726	16,570	16.8	This study
YaR2	12,824,206	1,883,363,892	2,247	341,531	2,242	324,212	16,571	19.6	This study
HaR3	38,863,726	5,759,994,672	27,857	4,166,865	27,817	4,160,749	16,569	251.1	This study
HaR2	39,932,022	5,989,803,300	39,417	5,764,437	39,411	5,763,387	16,569	347.8	Extracted from SRR11526798
LDN1	29,999,252	4,499,887,800	242,437	36,380,063	90,796	13,618,597	16,569	821.9	Extracted from SRR11526794

Table S3. Summary of the published mitogenomes used in phylogenetic analyses.

**Family**	**Species**	**Accession number**	**Mitogenome size (bp)**
Cobitidae	*Misgurnus anguillicaudatus*	DQ026434	16,565
	Cobitis sinensis	AY526868	16,553
Gastromyzontidae	*Sewellia lineolata*	AP011292	16,522
Nemacheilidae	*Triplophysa rosa*	JF268621	16,585
	Triplophysa robusta	KM406486	16,570
	Triplophysa orientalis	KJ631323	16,562
	Triplophysa angeli	MZ325251	16,569
	Triplophysa bleekeri	JX135578	16,568
	Triplophysa stewarti	KJ631324	16,567
	Triplophysa dorsalis	KT241024	16,572
	Triplophysa labiata	OQ559481	16,573
	Triplophysa dalaica	KY945353	16,569
	Triplophysa tenuis	KR052018	16,571
	Triplophysa bombifrons	KP297875	16,569
	Triplophysa strauchii	KT224366	16,590
	Triplophysa xichangensis	JQ663847	16,570
	Triplophysa stoliczkai	DQ026434	16,571

Table S4. Variable sites of the 13 PCGs in eight *T. dalaica* mitochondrial genomes

**Gene**	**CDS Length (bp)**	**No. Variable Sites (CDS)**	**Protein Length (AA)**	**No. Variable Sites (protein)**
ND1	975	182	324	15
ND2	1045	227	348	38
COX1	1551	175	516	1
COX2	691	61	230	0
ATP8	168	6	55	0
ATP6	684	92	227	5
COX3	784	90	261	2
ND3	349	51	116	7
ND4L	297	44	98	2
ND4	1382	238	460	21
ND5	1839	320	612	43
ND6	522	97	173	9
CYTB	1141	176	380	7

Table S5. The best partition scheme and substitution model by PartitionFinder 2.

**Subset**	**Best substitution model**	**Partition scheme**
1	GTR+I+G	COX2_pos1, COX1_pos1, ND6_pos1, ND4L_pos1, COX3_pos1, ND4_pos1, CYTB_pos1, ND1_pos1, ATP6_pos1, ND5_pos1, ND2_pos1, ND3_pos1
2	GTR+I+G	ATP6_pos2, ATP8_pos2, ATP8_pos1, ND5_pos2, ND2_pos2, COX2_pos2, ND4L_pos2, COX1_pos2, CYTB_pos2, COX3_pos2, ND6_pos2, ND4_pos2, ND1_pos2, ND3_pos2
3	GTR+I+G	ND2_pos3, ND3_pos3, CYTB_pos3, ND1_pos3, ND4_pos3, COX1_pos3, ND5_pos3, ND4L_pos3, ATP6_pos3, COX3_pos3, COX2_pos3, ATP8_pos3
4	GTR+G	ND6_pos3

Fig S1. Divergence time among mitogenomes in this study with MCMC approach. Bars at nodes exhibit 95% credible intervals of the divergence time estimates. The red dot indicates the placement of the calibration point.

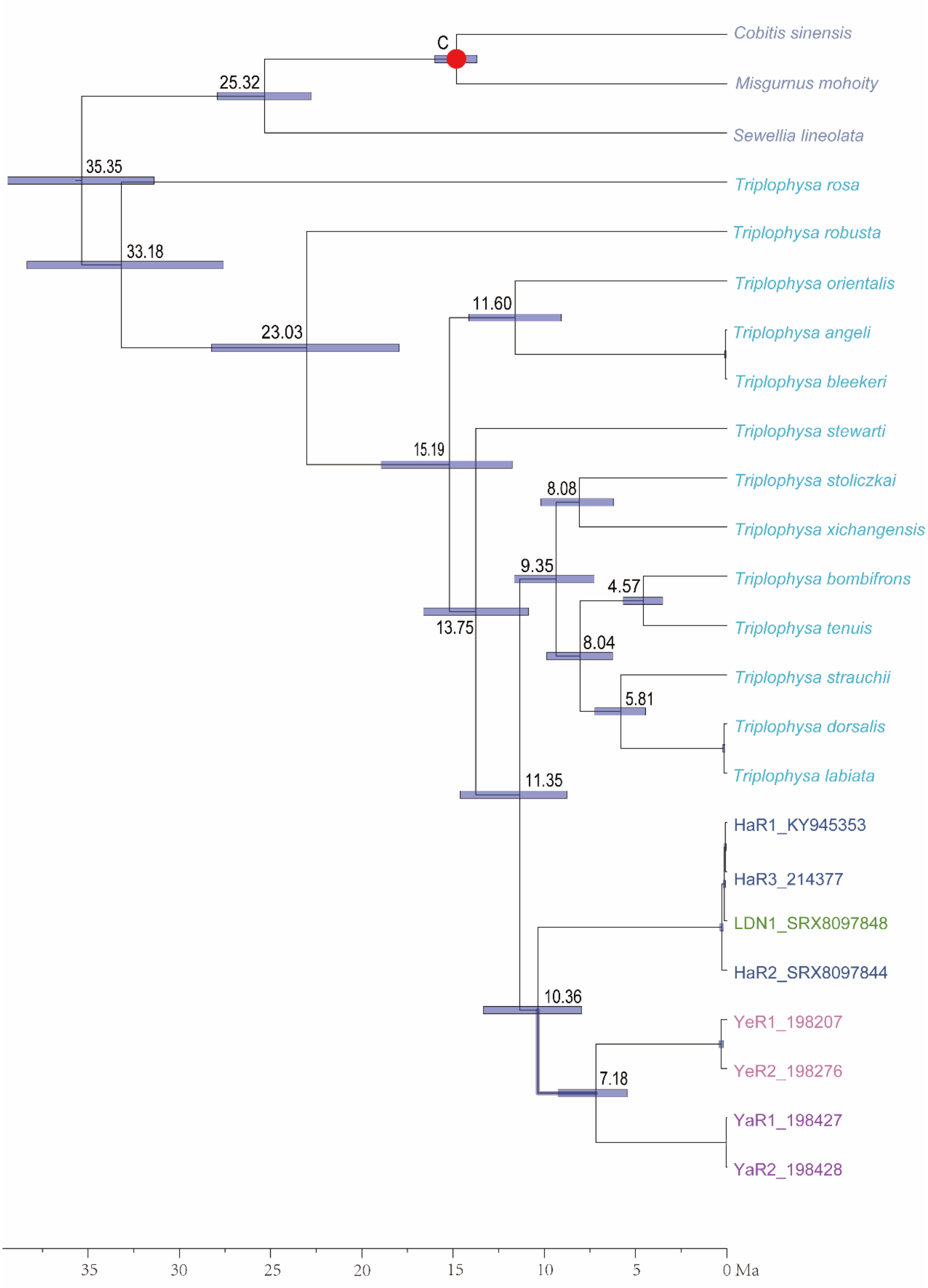

Supplement: Supplementary material 1 — Supplementary data [file zookeys-1197-043_article-116342__-s001.pdf]
